# LINC00623/miR-101/HRAS axis modulates IL-1β-mediated ECM degradation, apoptosis and senescence of osteoarthritis chondrocytes

**DOI:** 10.18632/aging.102801

**Published:** 2020-02-12

**Authors:** Guohua Lü, Lei Li, Bing Wang, Lei Kuang

**Affiliations:** 1 Department of Spine Surgery, The Second Xiangya Hospital, Central South University, Changsha 410011, Hunan, China

**Keywords:** osteoarthritis (OA), extracellular matrix (ECM), lncRNA LINC00623, miR-101, harvey rat sarcoma viral oncogene homolog (HRAS)

## Abstract

Chondrocyte apoptosis and extracellular matrix (ECM) degeneration have been implicated in the pathogenesis of osteoarthritis (OA). Based on previously reported microarray analysis, HRAS (Harvey rat sarcoma viral oncogene homolog), a member of the RAS protein family, was chosen as a potential regulator of OA chondrocyte apoptosis and ECM degradation. HRAS expression was downregulated in OA tissues, particularly in mild-OA tissues. HRAS overexpression partially attenuated IL-1β-induced OA chondrocyte apoptosis and ECM degradation. Similar to HRAS, the long non-coding RNA LINC00623 was downregulated in OA tissues. LINC00623 knockdown enhanced IL-1β-induced OA chondrocyte apoptosis and ECM degradation, which could be partially reversed by HRAS overexpression. It has been reported that lncRNAs act as ceRNAs of miRNAs to exert their function. Herein, miR-101 was predicted to bind to both LINC00623 and HRAS, which was further confirmed by luciferase reporter and RIP assays. LINC00623 competed with HRAS for miR-101 binding, therefore reducing the inhibitory effect of miR-101 on HRAS expression. More importantly, the effect of LINC00623 was partially eliminated by miR-101 inhibition. Overall, the LINC00623/miR-101/HRAS axis modulates OA chondrocyte apoptosis, senescence and ECM degradation through MAPK signaling, which might play a critical role in OA development.

## INTRODUCTION

Osteoarthritis (OA), a degenerative joint disease that is characterized by progressive degenerative alterations in the articular cartilage and other joint tissues [1, 2], chondrocyte hypertrophy and apoptosis [3, 4] is reported to be the fourth leading cause of disability [5, 6]. The pathology of OA can be controlled through the processing of both genetic and environmental information [2].

Chondrocyte apoptosis and senescence have been detected in OA cartilage, which is associated with extracellular matrix (ECM) degradation [7, 8] and participates in the initiation and development of OA [9–11]. Enhanced chondrocyte apoptosis has been reported to be significantly correlated with the severity of OA both *in vitro* and *in vivo* in studies of animals [12] and humans [13, 14]. Understanding the mechanism of OA chondrocyte apoptosis and senescence regulation may be of great clinical value for OA treatment.

Furthermore, the function of normal articular cartilage depends on the functional integrity of its ECM, which is rich in fibrillar collagens, especially type II collagen (Collagen II) [15]. During the pathology of OA, the balance between the synthesis and degradation of ECM components maintained by chondrocytes is disrupted, resulting in the structural and functional dysregulation of cartilage [16]. MMP13, a collagenase with substrate specificity that targets collagen for degradation [17], has been reported to preferentially cleave Collagen II and is thus considered a major contributor to OA cartilage degeneration.

During the past few decades, abrogation of epigenetic regulation has become evident in OA. Epigenetics enables tight control of gene expression at the transcriptional level, resulting in changes in chromatin 3D structure, and at the translational level (microRNAs (miRNAs), long noncoding RNAs (lncRNAs), mRNA editing and mRNA stability) affecting protein expression [18]. The deregulation and dysfunction of mRNAs [19–22], lncRNAs [23, 24] and miRNAs [25–28] in OA have been reported. Previously, Fu et al. [23] revealed that a total of 710 mRNAs were differentially expressed in OA tissues. In the present study, these differentially expressed genes were annotated in KEGG (Kyoto Encyclopedia of Genes and Genomes) pathway analysis to identify the key signaling pathway. The enrichment p-values were shown in the Supplementary Table 1. As shown in Supplementary Figure 1A, MAPK signaling, which is related to cell proliferation, contained the most altered genes (Supplementary Figure 1A). Furthermore, altered genes were subjected to protein-protein interaction analysis using the String database, and HRAS (Harvey rat sarcoma viral oncogene homolog) was chosen for further study due to its well-known role in proliferation, most commonly through Raf/ERK cascade signaling [29].

It has been widely recognized by several *in vitro* studies that the proinflammatory cytokine IL-1β can induce cartilage destruction in many cell types, including chondrocytes [30]. Herein, IL-1β-stimulated chondrocytes were used to assess the function of HRAS in cell apoptosis, senescence and ECM degradation. Next, the correlation between deregulated lncRNAs and HRAS was analyzed to investigate the molecular mechanism. Moreover, online tools were used to identify the miRNAs that could bind to both HRAS and selected lncRNAs, and the predicted targeting interaction and the related function were then verified. Overall, we provide a novel mechanism by which chondrocyte apoptosis, senescence and ECM degradation might be regulated in OA pathological progression from the perspective of the lncRNA-miRNA-mRNA regulatory network.

## RESULTS

### Screening and validation of HRAS expression in tissue samples

**Figure 1:**
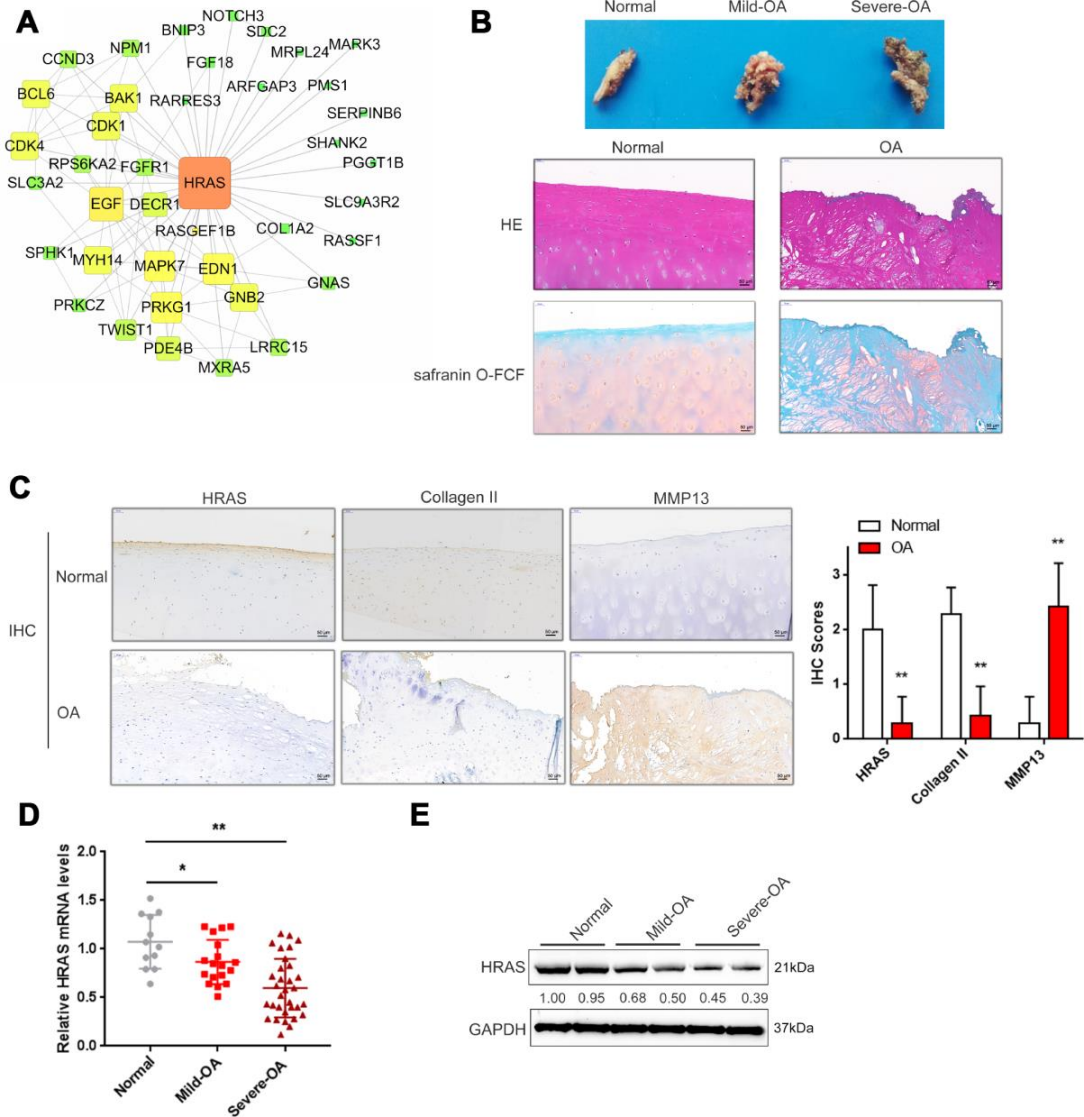
**Screening and validation of HRAS expression in tissue samples.** (**A**) The protein-protein interactions of the differentially expressed genes (both up and down regulated) were retrieved from the String (string-db.org) database and the subnetwork of interactions among MAPK pathway genes, including HRAS and EGF, was chosen and further visualized using Cytoscape software (version 3.4). (**B**) The macroscopic properties and pathological characteristics of normal and OA tissues were examined using H&E and Safranin O-FCF staining. (**C**) The localization of HRAS, Collagen II and MMP13 in normal and OA tissues examined using IHC staining. (**D**) The mRNA expression and protein levels (**E**) of HRAS in normal, mild OA and severe OA tissues were detected using real-time PCR and immunoblotting assays, respectively. The data are presented as mean ± SD of three independent experiments. *P<0.05, **P<0.01.

Based on the microarray results from Fu et al. [23], a total of 710 mRNAs were differentially expressed in osteoarthritis tissues (fold change>4, *P* < 0.01, 144 upregulated and 566 downregulated); these genes were annotated in a KEGG pathway analysis, and the major altered cellular signaling pathways were found to be the MAPK, PI3K/AKT and mTOR pathways (Supplementary Figure 1A). The fold-changes in MAPK pathway included changes in HRAS (fold-change = -4.08, P <0.01) and EGF (fold-change = -5.09, P < 0.01) as shown in Supplementary Figure 1B. Altered genes were subjected to protein-protein interaction analysis using the String database and visualized using Cytoscape (Supplementary Figure 1C, Figure 1A); the sub-network of interactions among MAPK pathway genes, including HRAS and EGF, were shown in Figure 1A. OA cartilage specimens were subjected to severity evaluation by the Kellgren and Lawrence (K/L) scoring system, and then divided into three groups: non-OA (normal), mild-OA, and severe-OA. The degenerative morphology of OA specimens of different severity was evaluated by macroscopic observation (Figure 1B, upper panel) and staining with H&E and Safranin O-FCF (Figure 1B, middle and bottom panels).

Activated chondrocytes play a key role in the OA process by producing matrix-degrading enzymes, including matrix metalloproteinase 13 (MMP13) [31]. The cartilage matrix, mainly composed of proteoglycans and Collagen II, is responsible for the maintenance of the normal cartilage structure. Degradation of the collagen network a critical event in OA may result in impaired tensile strength [32, 33]. Here, the protein levels of MMP13 and Collagen II in normal and OA cartilage tissues were examined using IHC. Consistent with previous studies, MMP13 levels were increased in OA tissues, while Collagen II was decreased, HRAS protein levels were also decreased (Figure 1C). Similarly, the mRNA expression of HRAS was reduced in OA tissue samples and further downregulated in mild- OA tissues (Figure 1D). The same trend was observed in immunoblotting assays (Figure 1E).

### HRAS inhibits IL-1β-induced chondrocyte apoptosis and senescence

**Figure 2:**
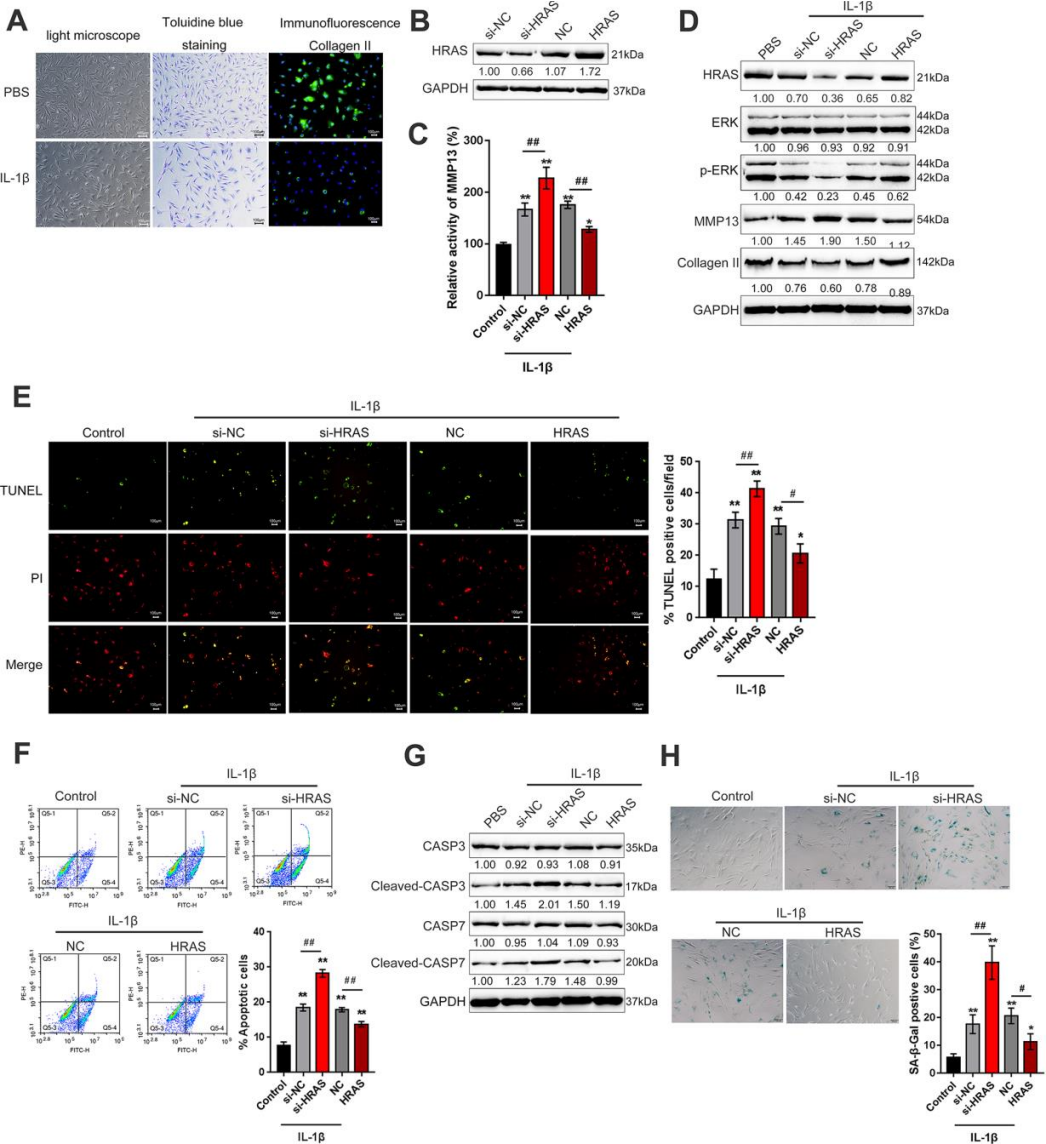
**HRAS inhibited IL-1β-induced chondrocyte apoptosis and senescence.** (**A**) Primary OA chondrocytes were isolated, treated with PBS or IL-1β and examined by light microscopy, toluidine blue staining and Collagen II immunofluorescence staining (IF). (**B**) HRAS knockdown or overexpression was achieved by transfection of si-HRAS or HRAS overexpressing vector into chondrocytes, respectively, and was confirmed using immunoblotting. Transfected chondrocytes were exposed to PBS or IL-1β and then examined for the MMP13 activity by a SensoLytes Plus 520 MMP13 assay kit (**C**), the protein levels of HRAS, ERK, p-ERK, MMP13 and Collagen II using immunoblotting (**D**), cell apoptosis using TUNEL assays (**E**) and Flow cytometer assays (**F**), the protein levels of Caspase 3, cleaved-Caspase 3, Caspase 7 and cleaved-Caspase 7 using immunoblotting (**G**), and the SA-β-Gal positive cells were determined by the SA-β-Gal staining (**H**). The data are presented as mean ± SD of three independent experiments. *P<0.05, **P<0.01, compared to control group; #*P*<0.05, ##*P*<0.01, compared to IL-1β + si-NC (negative control for si-HRAS) or IL-1β + NC vector (negative control for HRAS) group.

To assess the detailed function of HRAS in OA chondrocyte apoptosis and senescence, primary OA chondrocytes were isolated, treated with PBS or IL-1β and examined by light microscopy, toluidine blue staining and Collagen II immunofluorescence staining (IF). As shown in Figure 2A, OA chondrocytes were successfully isolated, and IL-1β treatment reduced the cell number and Collagen II density. The knockdown of HRAS was conducted by the transfection of si1/2/3-HRAS in OA chondrocytes and the transfection efficiency was verified by real-time PCR; si1-HRAS was selected as the siRNA for HRAS because of better transfection efficiency (Supplementary Figure 2D). OA chondrocytes were transfected with si-HRAS or an HRAS overexpressing vector to modulate HRAS expression, which was confirmed using immunoblotting (Figure 2B). Transfected chondrocytes were exposed to PBS or IL-1β treatment, and then examined for related indexes. IL-1β significantly induced the MMP13 activity; HRAS knockdown further enhanced, while HRAS overexpression attenuated IL-1β-induced MMP13 activity (Figure 2C). IL-1β treatment obviously reduced the protein levels of HRAS, p-ERK and Collagen II, while it increased MMP13 protein levels; HRAS knockdown further enhanced the effect of IL-1β treatment on the above proteins; however, HRAS overexpression partially attenuated the effect of IL-1β (Figure 2D). Next, TUNEL and flow cytometry assays were performed to examine cell apoptosis. Consistent with previous findings, both assays revealed that HRAS knockdown enhanced chondrocyte apoptosis, while HRAS overexpression attenuated IL-1β-induced chondrocyte apoptosis (Figure 2E–2F). Furthermore, alterations in alternations in apoptosis-related Caspase 3/7 protein levels were examined. IL-1β treatment increased the protein levels of cleaved-Caspase3/7, which was further enhanced by HRAS knockdown but partially attenuated by HRAS overexpression (Figure 2G). Moreover, IL-1β significantly increased senescence-associated β-galactosidase (SA-β-Gal) staining positive cells; HRAS knockdown further increased, while HRAS overexpression decreased IL-1β-induced increase in SA-β-Gal positive cells (Figure 2H).

### LINC00623 upregulates HRAS to suppress IL-1β-induced chondrocyte apoptosis and senescence

**Figure 3:**
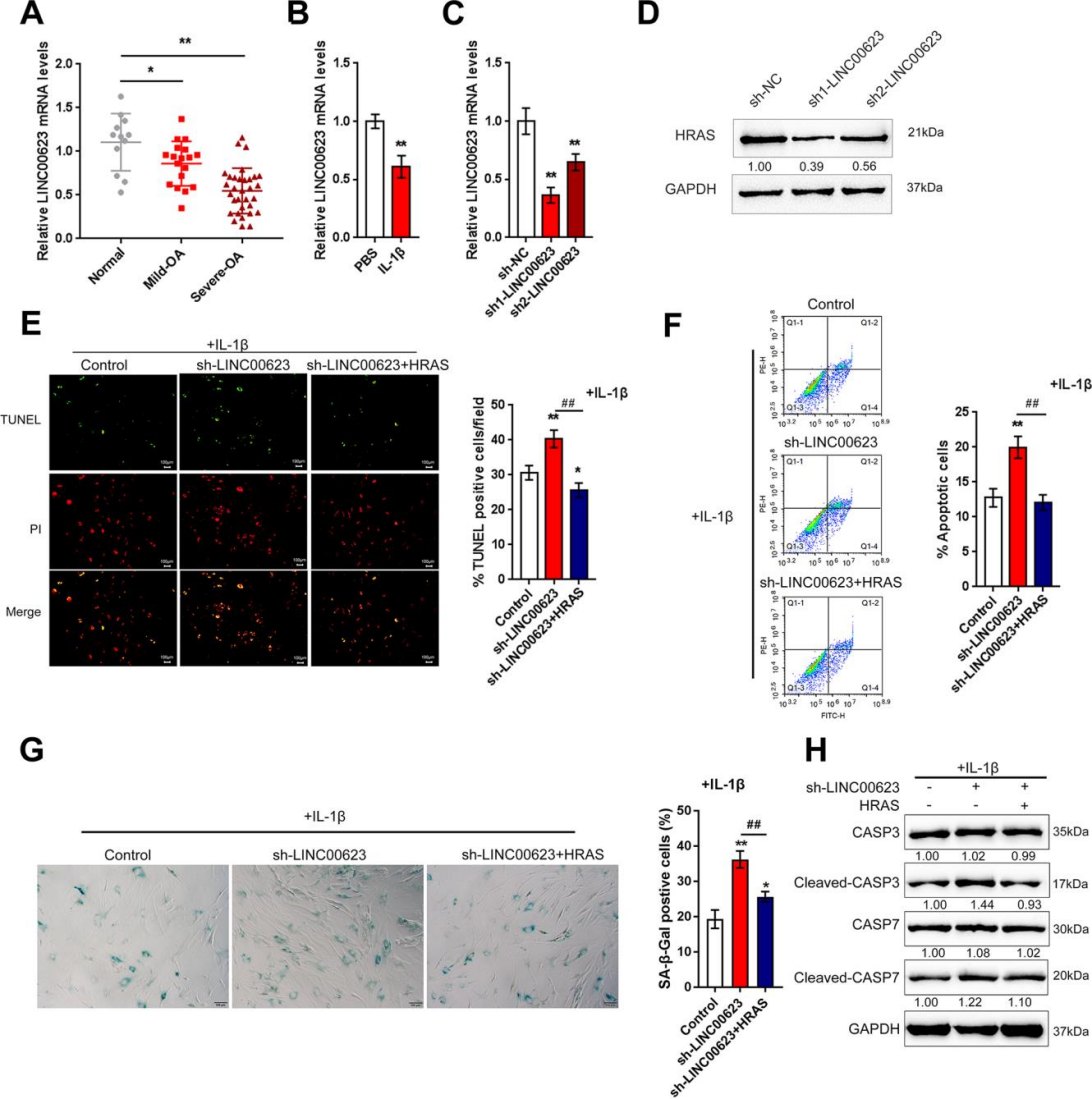
**LINC00623 upregulates HRAS to suppress IL-1β-induced chondrocyte apoptosis and senescence.** (**A**) LINC00623 expression in normal, mild OA and severe OA tissues was examined using real-time PCR assays. (**B**) OA chondrocytes were treated with PBS or IL-1βand then examined for LINC00623 expression using real-time PCR. (**C**) LINC00623 knockdown was achieved by transfection of sh1-LINC00623 or sh2-LINC00623, as confirmed using real-time PCR. (**D**) OA chondrocytes were cotransfected with sh1/2-LINC00623 and the HRAS vector in the presence or absence of IL-1β treatment and the protein levels of HRAS. (**E**, **F**) The cell apoptosis was examined using TUNEL and flow cytometer assays. (**G**) The SA-β-Gal positive cells were determined by the SA-β-Gal staining. (**H**) The protein levels of Caspase 3, cleaved-Caspase 3, Caspase 7 and cleaved-Caspase 7 were examined using immunoblotting. The data are presented as mean ± SD of three independent experiments. *P<0.05, **P<0.01, compared to control group; #*P*<0.05, ##*P*<0.01, compared to sh-LINC00623 group.

In addition to deregulated mRNAs, lncRNAs have also been reported to be differentially expressed in OA and normal cartilage tissues [23]. To investigate the mechanism by which HRAS modulates IL-1β-induced chondrocyte apoptosis and senescence, the correlation between HRAS and downregulated lncRNAs in OA tissues [23] was analyzed by Spearman’s rank correlation analysis. As shown in Supplementary Table 2 and Supplementary Figure 2A, a total of 11 lncRNAs were strongly positively correlated with HRAS, and LINC00623 was the most relevant lncRNA. A small sample size verification was performed to detect the expression of these lncRNAs in normal and OA tissues after online data retrieval and correlation analysis (Supplementary Figure 2B). The results demonstrated that LINC00623 expression was dramatically reduced in OA tissues (Supplementary Figure 2C). Similar to HRAS, LINC00623 expression was more downregulated in severe OA tissues (Figure 3A). LINC00623 expression was inhibited by IL-1β treatment (Figure 3B).

Next, LINC00623 downregulation was achieved by transfecting OA chondrocytes with sh1-LINC00623 or sh2-LINC00623 and was confirmed using real-time PCR (Figure 3C); LINC00623 expression was more downregulated by sh1-LINC00623. As shown in Figure 3D, HRAS protein was reduced by sh1/2-LINC00623 transfection and more reduced by sh1-LINC00623; thus, sh1-LINC00623 was selected for further experiments. OA chondrocytes were cotransfected with sh-LINC00623 and HRAS overexpression plasmid upon IL-1β treatment, and then examined for cell apoptosis and apoptosis-related Caspase 3/7 protein levels. IL-1β-induced chondrocyte apoptosis could be even enhanced by LINC00623 knockdown, which was partially attenuated by HRAS overexpression (Figure 3E–3F). Consistently, IL-1β-induced chondrocyte senescence could be further enhanced by LINC00623 knockdown while partially attenuated by HRAS overexpression (Figure 3G). Moreover, LINC00623 knockdown increased the protein levels of cleaved-Caspase3/7, while HRAS overexpression reversed this enhancement (Figure 3H). The above findings suggest that LINC00623 knockdown enhanced IL-1β-induced OA chondrocyte apoptosis and senescence while HRAS overexpression reduced this the enhancement.

### miR-101 is involved in LINC00623 regulating HRAS

**Figure 4:**
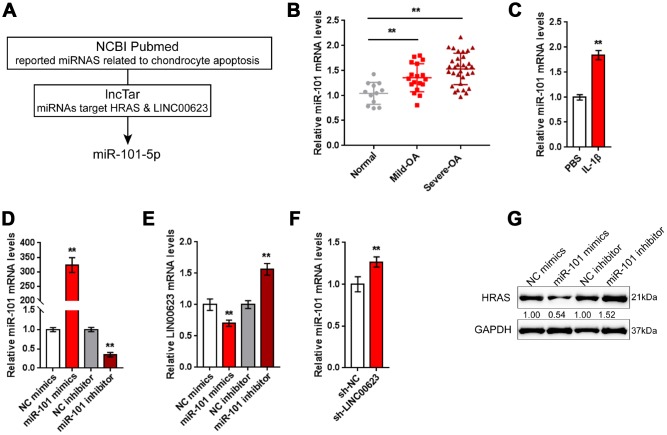
**miR-101 is involved in LINC00623 regulating HRAS.** (**A**) Previously reported miRNAs related to chondrocyte apoptosis were analyzed; the online tool LncTar was used to identify miRNAs that could be targeted by LINC00623; and miR-101-5p was selected. (**B**) The expression of miR-101 in normal, mild OA and severe OA tissues was examined using real-time PCR. (**C**) OA chondrocytes were treated with PBS or IL-1β and then examined for miR-101 expression using real-time PCR. (**D**) miR-101 expression was regulated by transfection of miR-101 mimics or miR-101 inhibitor, and was confirmed using real-time PCR. (**E**) LINC00623 mRNA expression in response to miR-101 overexpression and miR-101 inhibition was examined using real-time PCR. (**F**) miR-101 expression in response to LINC00623 knockdown was examined using real-time PCR. (**G**) HRAS protein levels in response to miR-101 overexpression and miR-101 inhibition were examined using immunoblotting. The data are presented as mean ± SD of three independent experiments. **P<0.01.

LncRNAs can exert their biological effects by serving as competing endogenous RNAs (ceRNAs) to inhibit miRNA expression, therefore restoring the expression of miRNA downstream targets [34, 35]. Since LINC00623 positively regulates HRAS expression in OA chondrocytes, in the present study, the online tools miRcode [36], RNA hybrid [37] and LncTar [38] were used to identify miRNAs that might be related to both LINC00623 and HRAS. As reported by previous studies, miR-101 inhibition can protect OA cartilage from degeneration by regulating ECM-related genes [39], and is predicted to bind LINC00623 and HRAS; therefore, miR-101 was chosen for further study (Figure 4A).

Contrary to LINC00623 and HRAS expression, the expression of miR-101 was remarkably upregulated in OA tissues, particularly in mild OA tissues (Figure 4B) and could be obviously promoted by IL-1β treatment (Figure 4C). To evaluate the precise function of miR-101 in OA, OA chondrocytes were transfected with miR-101 mimics or miR-101 inhibitor to achieve miR-101 expression, which was confirmed using real-time PCR (Figure 4D). In chondrocytes, miR-101 and LINC00623 negatively regulated each other (Figure 4E–4F). Moreover, miR-101 negatively modulated the protein level of HRAS (Figure 4G).

### LINC00623 competes with HRAS for miR-101 binding

**Figure 5:**
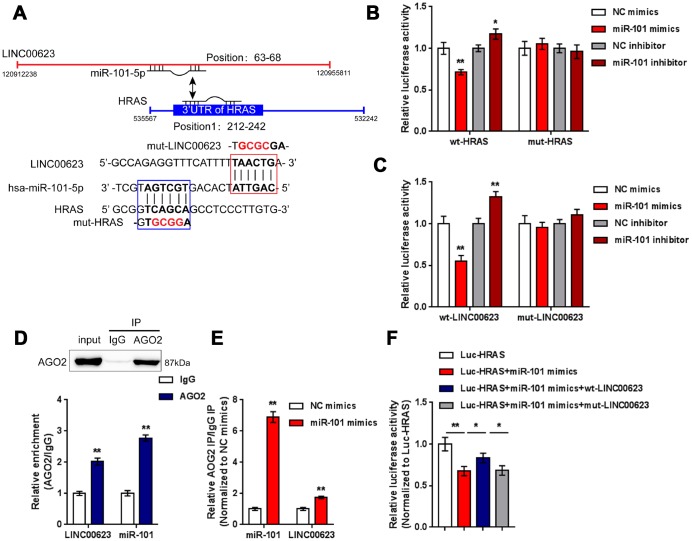
**LINC00623 competes with HRAS for miR-101 binding.** (**A**) Four different types of luciferase reporter gene vectors were constructed: a wt-LINC00623 or a wt-HRAS 3′-UTR containing the wild-type miR-101 binding site, and a mut-LINC00623 or a mut-HRAS 3′-UTR containing the mutated miR-101 binding site. (**B**, **C**) These vectors were cotransfected into HEK293 cells with miR-101 mimics or miR-101 inhibitor; the luciferase activity was examined. (**D**, **E**) RIP assays were performed to confirm the predicted binding between LINC00623 and miR-101 using an anti-AGO2 antibody. (**F**) the HRAS luciferase reporter vector, miR-101 mimics and wt- or mut-LINC00623 vector were cotransfected into HEK293 cells, the luciferase activity was examined. The data are presented as mean ± SD of three independent experiments. *P<0.05, **P<0.01.

After confirming the regulation of LINC00623, miR-101 and HRAS, the direct binding of miR-101 to LINC00623 and to HRAS, as predicted by online tools was verified. Two types of luciferase reporter gene vectors were constructed: wt- vectors containing wild-type HRAS 3′-UTR or LINC00623 fragments, and mut- vectors containing HRAS 3′-UTR or LINC00623 fragments with mutated predicted binding sites (Figure 5A). HEK293 cells were cotransfected with the above reporter vectors and miR-101 mimics or miR-101 inhibitor then examined for luciferase activity. The luciferase activity of the wild-type vectors (wt-HRAS 3′-UTR or wt-LINC00623) was significantly reduced by miR-101 mimics and increased by miR-101 inhibitor; after mutating the putative binding sites, the changes in luciferase activity were eliminated (Figure 5B–5C). Afterwards, RIP assays were performed with an anti-AGO2 antibody to confirm direct binding. In the AGO2 precipitate, the enrichment of LINC00623 and miR-15 was significantly higher than that of IgG (Figure 5D). In miR-101 mimic-transfected HEK293 cells, the relative enrichment (AGO2 IP/IgG IP) of miR-101 and LINC00623 was significantly higher than that of the NC mimics group (Figure 5E), indicating that LINC00623 could bind to miR-101 through AGO2 (RNA-induced silencing complex catalytic component). To verify the competitive binding of LINC00623 and HRAS to miR-101, wild-type HRAS 3′-UTR, miR-101 mimics and the wild-type or mutant-type LINC00623 vector were cotransfected into HEK293 cells. The luciferase activity of wild-type HRAS 3′-UTR was reduced by miR-101 mimics but partially restored by the wild-type LINC00623 vector, when co-transfected with mut-LINC00623, the luciferase activity of wild-type HRAS 3′-UTR reporter was again inhibited by miR-101 (Figure 5F), suggesting that LINC00623 competes with HRAS for miR-101 binding, therefore impairing the inhibitory effect of miR-101 on HRAS expression.

### The LINC00623/miR-101/HRAS axis modulates apoptosis, senescence and ECM degradation in OA chondrocytes through the MAPK signaling pathway

**Figure 6:**
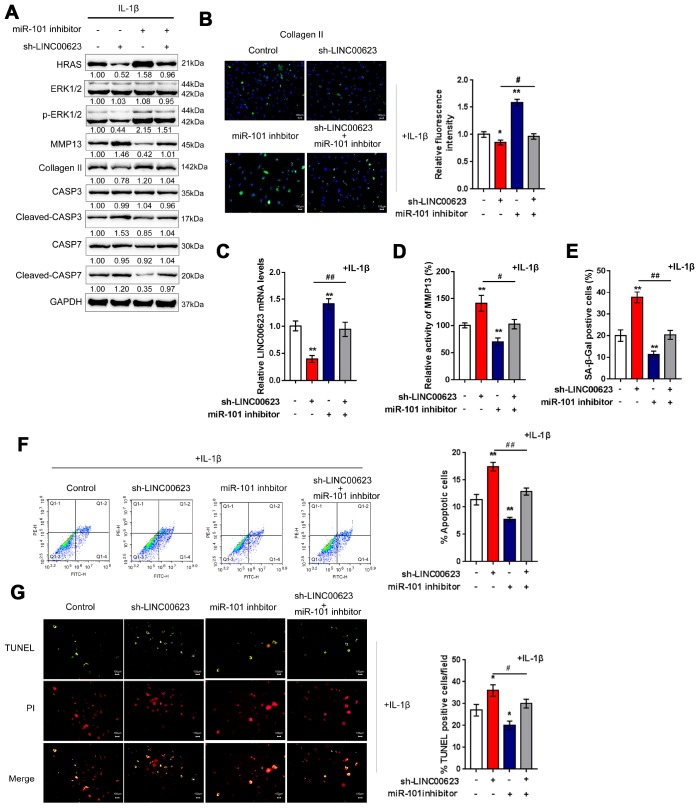
**LINC00623/miR-101/HRAS axis modulates the apoptosis, senescence and ECM degradation in OA chondrocytes through MAPK signaling pathway.** Chondrocytes were co-transfected with miR-101 inhibitor and sh-LINC00623 and expose to IL-1β stimulation; the protein levels of HRAS, ERK, p-ERK, MMP13, Collagen II, Caspase 3, cleaved-Caspase 3, Caspase 7 and cleaved-Caspase 7 were examined using immunoblotting (**A**); the localization of Collagen II was examined using IF (**B**); the expression levels of LINC00623 was determined by real-time PCR (**C**); the activity of MMP-13 was determined by a SensoLytes Plus 520 MMP13 assay kit (**D**); the SA-β-Gal positive cells were determined by the SA-β-Gal staining (**E**); the cell apoptosis was examined using flow cytometry (**F**) and TUNEL (**G**). The data are presented as mean ± SD of three independent experiments. *P<0.05, **P<0.01, compared to control group; #*P*<0.05, ##*P*<0.01, compared to sh-LINC00623 group.

OA chondrocytes were cotransfected with si-LINC00623 and miR-101 inhibitor in the presence of IL-1β stimulation, and then examined for ECM degradation-, apoptosis- and MAPK signaling-related proteins. As shown in Figure 6A, the protein levels of HRAS, p-ERK1/2, and Collagen II were reduced by LINC00623 knockdown and increased by miR-101 inhibition, while MMP13 and cleaved-Caspase3/7 proteins were increased by LINC00623 knockdown and increased by miR-101 inhibition. Moreover, the effect of LINC00623 knockdown on the above proteins was significantly attenuated by miR-101 inhibition (Figure 6A). IF assays further confirmed that Collagen II levels were significantly reduced by LINC00623 knockdown and increased by miR-101 inhibition, and the effect of LINC00623 knockdown was significantly attenuated by miR-101 inhibition (Figure 6B). Under the same conditions, sh-LINC00623 infection significantly inhibited, while miR-101 inhibitor transfection promoted the expression of LINC00623; the effects of sh-LINC00623 on LINC00623 expression were partially reversed by miR-101 inhibitor (Figure 6C). Consistently, LINC00623 knockdown increased, while miR-101 inhibition decreased the MMP13 activity (Figure 6D) and SA-β-Gal positive cells (Figure 6E); the effects of LINC00623 knockdown were significantly reversed by miR-101 inhibition (Figure 6D–6E). Furthermore, TUNEL and flow cytometry assays revealed that IL-1β-induced OA chondrocyte apoptosis could be enhanced by LINC00623 knockdown but reduced by miR-101 inhibition, and the effect of LINC00623 knockdown was partially attenuated by miR-101 inhibition (Figure 6F–6G). The above data indicate that the LINC00623/miR-101/HRAS axis modulates OA chondrocyte apoptosis, senescence and ECM degradation through MAPK signaling.

### The expression of IL-1β and the correlation of LINC00623, miR-101, HRAS and IL-1β in tissue samples

**Figure 7:**
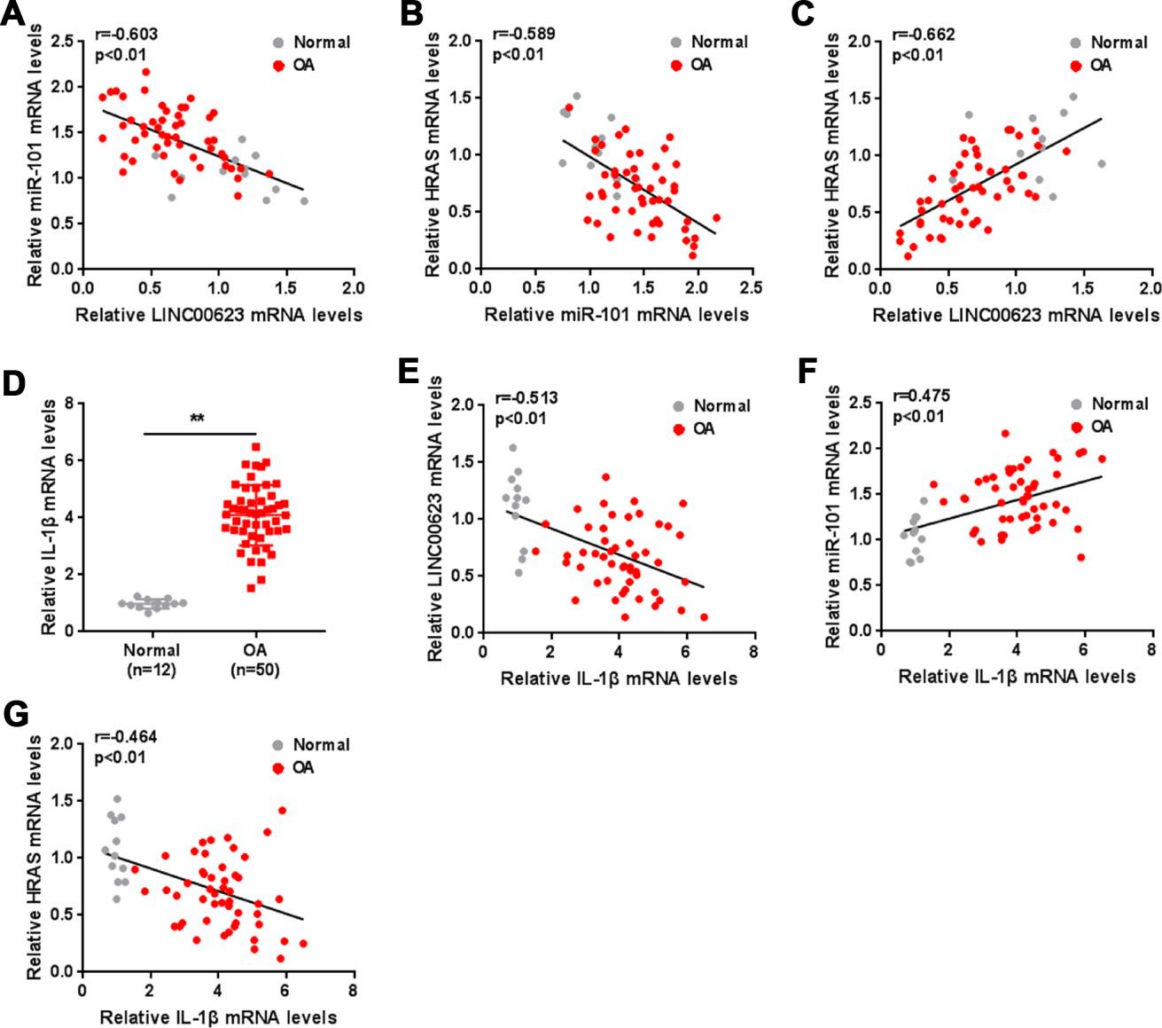
**The expression of IL-1β and the correlation of LINC00623, miR-101, HRAS and IL-1β in tissue samples.** (**A**–**C**) The correlation of LINC00623, miR-101 and HRAS expression was analyzed using Spearman’s rank correlation analysis. (**D**) The mRNA expression of IL-1β in normal and OA tissues were detected using real-time PCR. The data are presented as mean ± SD of three independent experiments. **P<0.01. (**E**–**G**) The correlation of IL-1β with LINC00623, miR-101 and HRAS expression, respectively, was analyzed using Spearman’s rank correlation analysis.

As a further confirmation of these findings, the correlation of LINC00623, miR-101 and HRAS expression in tissue samples was analyzed. miR-101 was negatively correlated with LINC00623 and HRAS (Figure 7A–7C). IL-1β mRNA expression was significantly upregulated in OA cartilage tissues (Figure 7D), negatively correlated with LINC00623 and HRAS expression, and positively correlated with miR-101 expression (Figure 7E–7G).

## DISCUSSION

In the present study, HRAS was selected as a potential regulator of OA chondrocyte apoptosis, senescence and ECM degradation based on previous microarray analysis and protein-protein interaction analysis. HRAS overexpression attenuated IL-1β-induced OA chondrocyte apoptosis, senescence and ECM degradation. Of the downregulated lncRNAs in OA tissues reported in the same microarray analysis, LINC00623 was the most relevant to HRAS. Consistent with its correlation with HRAS, LINC00623 knockdown enhanced IL-1β-induced MMP13 activity, OA chondrocyte senescence, apoptosis, and ECM degradation. As predicted by online tools and further validated by luciferase reporter gene and RIP assays, miR-101 bound to both LINC00623 and HRAS, and LINC00623 competed with HRAS for miR-101 binding to reduce the suppressive effect of miR-101 on HRAS expression. The effect of LINC00623 knockdown on OA chondrocytes was significantly attenuated by miR-101 inhibition.

HRAS is one of the RAS proteins which are small GTPases that cycle between inactive guanosine diphosphate (GDP)-bound and active guanosine triphosphate (GTP)-bound conformations [40]. The RAS signaling pathway can be activated by a series of cellular stimuli, and can modulate a complicated pathway network that includes RAF-MEK-ERK signaling, the PI3K pathway and the effector family of exchange factors for RAL small GTPases [41]. However, HRAS is regarded as an oncogene and is well known for its proliferative role in cancers. While, dysregulation of HRAS and related pathways is frequently observed in several cancers; its role in OA has not been investigated. According to Fu et al. [23], HRAS expression was significantly downregulated in OA tissues. Combined with KEGG and String analyses, HRAS might be a potential regulator of OA chondrocyte proliferation. Accordingly, downregulated expression of HRAS in OA tissues, particularly mild OA tissues was observed, along with the downregulation of Collagen II and upregulation of MMP13, suggesting that HRAS might also affect ECM degradation in OA chondrocytes. Consistent with its expression pattern in OA tissues, HRAS overexpression significantly attenuated IL-1β-induced OA ECM degradation, chondrocyte apoptosis, and chondrocyte senescence. In addition, HRAS overexpression reduced the protein levels of active Caspase 3/7 induced by IL-1β stimulation, suggesting that HRAS might modulate Caspase-dependent chondrocyte apoptosis.

OA pathology is a complicated process that can be modulated by both genetic and environmental factors. In addition to mRNA deregulation, differentially expressed lncRNAs in OA tissues were also proposed by Fu et al. [23]. Among these deregulated lncRNAs, LINC00623 was the most relevant to HRAS expression; moreover, LINC00623 expression was also downregulated in OA tissues, particularly in mild OA tissues, the same as HRAS. More remarkably, lncRNAs have been reported to be related to cell senescence and age-related degeneration of articular cartilage in OA. Two hub lncRNAs, CRNDE and LINC00152, have been regarded as the key lncRNAs in age-related degeneration of articular cartilage through comprehensive and integrative analysis [42]. Therefore, LINC00623 might also act on chondrocyte senescence in a HRAS-related way. In the present study, LINC00623 knockdown enhanced the effect of IL-1β stimulation on chondrocyte apoptosis, senescence, and ECM degradation, while this effect could be partially eliminated by HRAS overexpression, suggesting that LINC00623 might cooperate with HRAS to modulate chondrocyte apoptosis, senescence and ECM degradation.

Regarding the molecular mechanism, lncRNAs can serve as ceRNAs of miRNAs to impair the suppressive effect of miRNAs on the gene expression of downstream targets, therefore rescuing downstream gene expression [34]. An upregulated lncRNA, namely lncRNA-CIR, was found to acts as a ceRNA to compete with MMP-13 for miR-27 binding, therefore inhibiting miR-27 expression and upregulating MMP-13 expression, finally promoting ECM degradation of chondrocytes in OA [43]. Similar to LINC00623 expression, the expression of lncRNA UFC1 was also downregulated in OA tissues, and by actingas a “sponge” of miR-34a, lncRNA UFC1 promoted OA chondrocyte proliferation [44]. It’s worth noting that, miRNAs have also been reported to play a critical role in regulating cell senescence. miR-199a-3p and miR-193b expression is upregulated with age and may be involved in chondrocyte senescence by downregulating anabolic factors such as type 2 collagen, aggrecan, and SOX9 [45]. Another miRNA, miR-34a, has been reported to noticeably inhibit the expression of DLL1, trigger cell death and senescence, suppress proliferation, and prevent scratch assay wound closure in rat chondrocytes and chondrosarcoma cells, therefore facilitating the development of OA [46]. Therefore, we further investigated miRNAs that might modulate OA chondrocyte senescence and apoptosis in a LINC00623 and HRAS-related way. in the present study, online tools were used to screen for miRNAs that might bind to both LINC00623 and HRAS. Among the candidate miRNAs, miR-101 silencing has been reported to protect cartilage from degeneration [39]. Herein, miR-101 expression was remarkably increased in OA tissues, particularly in mild OA tissues; moreover, LINC00623 and miR-101 negatively regulated each other, suggesting the potential role of miR-101 in OA chondrocytes.

As further confirmed, miR-101 could bind to both LINC00623 and HRAS; LINC00623 competed with HRAS for miR-101 binding, thus reducing the suppressive effect of miR-101 on the expression of its downstream target, HRAS. More importantly, although its role in OA has rarely been reported, miR-101 is well-known for its tumor suppressive function. By promoting cancer cell apoptosis, miR-101 can serve as a tumor suppressor [47–49]. In nasopharyngeal carcinoma cells, miR-101 exerted its apoptosis-promoting function by inhibiting the Ras/Raf/MEK/ERK signaling pathway [47]. Consistently, miR-101 inhibition significantly attenuated IL-1β-induced chondrocyte apoptosis, senescence, as well as ECM degradation; moreover, the effect of LINC00623 knockdown on chondrocytes was significantly eliminated by miR-101 inhibition, further confirming that LINC00623/miR-101/HRAS axis modulates OA chondrocyte apoptosis, senescence, and ECM degradation through MAPK signaling.

In tissue samples, miR-101 expression was negatively correlated with LINC00623 and HRAS and positively correlated with IL-1β expression, while LINC00623 and HRAS expression were both negatively correlated with IL-1β expression and positively correlated with each other, further confirming that LINC00623 acted as a ceRNA to compete with HRAS for miR-101 binding, thus suppressing its effect on IL-1β-induced OA chondrocyte apoptosis, senescence and ECM degradation.

In conclusion, the LINC00623/miR-101/HRAS axis modulates OA chondrocyte apoptosis, senescence and ECM degradation through MAPK signaling. As a possible further direction of development of this study, there might be more lncRNA-miRNA-mRNA interactions during OA pathology. Since miRNAs could simultaneously target one or multiple downstream targets, multiple signaling pathways might be involved, and should be validated in future study.

Notably, autophagy is a protective mechanism in normal cartilage for maintaining cell homeostasis by adjusting cell metabolism to nutrient supply and removing damaged organelles. In cartilage, aging-related loss of autophagy leads to cell death and OA, while stimulation of autophagy exerts protective effects on cartilage deterioration. A series of miRNAs are involved in the progression of chondrocyte autophagy and are connected with numerous factors and pathways [50, 51]. For example, miR-335-5p was significantly downregulated in OA chondrocytes and the overexpression of miR-335-5p in human OA chondrocytes led to remarkable increases in viability and autophagy-related factors, and a reduction in inflammatory mediators; treatment with the autophagy inhibitor 3-MA restored the expression of inflammatory mediators [52]. miR-128a-induced Atg12 loss repressed chondrocyte autophagy to aggravate OA progression [53]. Thus, we speculated that the LINC00623/miR-101/HRAS axis might also regulate autophagy in OA chondrocytes, which needs further experimental investigation.

## MATERIALS AND METHODS

### Cartilage acquisition and assessment

Normal cartilage tissues were obtained from non-OA traumatic amputees, and degenerated cartilage tissues were obtained from OA patients who had undergone total knee replacement under the approval of the institutional review board and ethics committee of The Second Xiangya Hospital. All experiments were conducted in accordance with the approved guidelines. Written informed consents were obtained from all subjects. The cartilage tissues were assessed with hematoxylin- eosin (HE) staining, safranin-O staining, Immunohistochemistry, and a modified Mankin grading system in previous study [54–56].

### Primary OA chondrocyte isolation, identification and culture conditions

Primary OA chondrocytes were isolated and cultured according to a previous study [54–56]. Degenerated cartilage tissue samples were minced into pieces of less than 1 mm^3^, followed by digestion at 37 °C with 0.15% collagenase II (Invitrogen, Carlsbad, CA, USA) for 5 - 6 h with stirring every 20 min after 2 h. Chondrocytes were isolated after centrifugation and cultured in DMEM-F12 containing 10% fetal bovine serum (FBS) and antibiotics for 5 - 7 days before use. Isolated chondrocytes were identified by light microscopy, toluidine blue staining and type II collagen immunofluorescence staining (IF).

HRAS expression was achieved by transfection of si-HRAS or HRAS overexpressing vector (GeneCopoecia, Guangzhou, China); LINC00623 knockdown was achieved by transfection of sh1-LINC00623 or sh2-LINC00623 (GeneCopoecia). The modulation of miR-101 was achieved by transfection of miRNA mimics or miRNA inhibitors (Genepharma, Shanghai, China) with the help of Lipofectamine 2000 (Invitrogen).

### Hematoxylin and eosin (H&E) and Safranin O or Fast Green (FCF) staining

Normal and OA cartilage tissues were fixed in 10% zinc-buffered formalin overnight and then processed for paraffin embedding and sectioning. Sections of 4 μm were deparaffinized, rehydrated, and then stained using an H&E staining kit (Beyotime, Shanghai, China) or Safranin O-FCF (OriGene Technologies, Inc., Rockville, MD, USA) according to the protocols.

### Immunohistochemistry (IHC) staining

Immunohistochemistry staining was performed using the Vectastain Universal Elite ABC Kit (Vector Laboratories, Burlingame, CA, USA). Normal and OA cartilage sections were incubated at 4 °C overnight with primary antibodies against HRAS (Cat# AP7764C, Abgent, San Diego, CA, USA), Collagen II (ab34712, Cambridge, MA, USA), and MMP13 (ab39012, Abcam). The IHC scores were as follows: 0 = negative; 1 = weak; 2 = moderate; 3 = strong.

### TUNEL staining

Apoptotic cells were determined using a terminal deoxynucleotidyl transferase-mediated dUTP nick end labeling (TUNEL) assay and costaining with propidium iodide (PI) [57]. The TUNEL procedure was performed using an *in-situ* cell death detection kit (Roche, Indianapolis, IN, USA). TUNEL-positive OA chondrocytes were counted under a microscope under double-blinded conditions. The ratio of TUNEL-positive OA chondrocyte cells to the total number of OA chondrocytes was then calculated.

### Immunofluorescence staining

For the detection of Collagen II, cells (1×10^5^ per well) were seeded in 6-well glass bottom plates. After the cells were treated, they were fixed in 4% paraformaldehyde for 30 min and then permeabilized with 0.2% Triton X-100 for 15 min. Nonspecific binding sites were blocked with 1% BSA in PBS for 2 h. Then, the cells were treated with primary antibody specific to Notch-1(1:200, diluted in 1% BSA) overnight at 4 °C. Thereafter, the cells were incubated with TRITC-conjugated secondary antibody (Beyotime, China) for 1 h in the dark. DAPI (Beyotime, China) was used to stain nuclei before capturing images. The images were acquired using a fluorescence microscope (Nikon, Japan). The red fluorescence indicates Notch-1 expression, and the blue fluorescence indicates nuclei.

### Flow cytometer assay

Quantification of apoptotic cells was performed with an Annexin V-FITC apoptosis detection kit (Keygen, China). Briefly, the cell samples were harvested with 0.25% trypsin without EDTA 48 h after transfection and then washed twice with ice-cold PBS and resuspended in 500 μl binding buffer. Then, the cells were incubated with 5 μl Annexin V-FITC specific antibodies and 5 μl propidium iodide (PI) incubated for 15-20 min in the dark and detected by BD Accuri C6 flow cytometry (BD, USA) with the excitation wavelength of Ex = 488 nm and emission wavelength of Em = 530 nm. Each experiment was repeated three times in triplicate.

### Measurement of MMP13 activity

To detect the activity of MMP13 in culture media, the media were collected and centrifuged at 10,000 rpm for 5 min. Then, 25 μl of the supernatant was used to measure MMP13 activity with a a SensoLytes Plus 520 MMP13 assay kit (AnaSpec, San Jose, CA, USA) according to the manufacturer’s instructions. The fluorescence signal is monitored at Ex/Em=490 nm/520 nm by GloMax®-Multi+ Detection System (Promega, Madison, WI, USA).

### Senescence-associated β-galactosidase (SA-β-Gal) activity

The positive blue staining of SA-β-Gal has been used as a biomarker of cellular senescence [58]. To detect SA-β-Gal staining, we washed cells in subconfluent cultures with PBS, fixed them for 3–5 min in 3.7% formaldehyde, and then washed the cells again with PBS. Cells were incubated overnight at 37°C in the CO_2_-free atmosphere with SA-β-Gal stain solution (1 mg/ml X-gal, 40 mM citric acid/sodium phosphate, pH 6.0, 5 mM potassium ferrocyanide, 5 mM potassium ferricyanide, 150 mM NaCl, 2 mM MgCl_2_). Positive staining appeared after 2–4 h and was evaluated after 12–14 h. Five hundred cells were scored using light microscopy to dertect SA-β-Gal positive cells.

### Immunoblotting

The protein levels of HRAS, ERK1/2, p-ERK1/2, MMP13, Collagen II, Caspase-3, cleaved-Caspase-3, Caspase-7 and cleaved-Caspase-7 were examined by immunoblotting. Target cells were lysed using RIPA buffer with 1% PMSF, and the proteins were extracted and analyzed for protein concentration using the bicinchoninic acid (BCA) protein assay kit (Beyotime Institute of Biotechnology, China). Proteins were then loaded onto SDS-PAGE minigel and transferred onto PVDF membranes. Thereafter, the membrane was probed with the following antibodies: anti-HRAS (ab140640, Abcam), anti-ERK1/2 (ab 184699, Abcam), anti-p-ERK1/2 (ab50011, Abcam), anti-MMP13 (ab39012, Abcam), anti-Collagen II (ab34712, Abcam), anti-Caspase-3 (ab13847, Abcam), anti-cleaved-Caspase-3 (ab2302, Abcam), anti-Caspase-7 (ab69540, Abcam) and cleaved-Caspase-7 (ab2323, Abcam) and anti-GAPDH (ab8245, Abcam) at 4°C overnight. Thereafter, the blots were incubated with HRP-conjugated secondary antibody (1:5000). Signal visualization was conducted by ECL Substrates (Millipore, MA, USA) using GAPDH as an endogenous protein for normalization. The gray intensity analysis was performed using ImageJ software (NIH).

### Real-time PCR

Total RNA was extracted from targeted tissues or cells using Trizol reagent (Invitrogen, CA, USA) and then treated with DNase I (Invitrogen, USA) according to the manufacturer’s instructions. Synthesis of the first strand (cDNA) was performed using oligo (dT) 20 and Superscript II reverse transcriptase (Invitrogen, USA). The expression of mRNA was detected by SYBR green PCR Master Mix (Qiagen) using GAPDH as an endogenous control. The expression of miRNA was examined by a Hairpin-it TM miRNAs qPCR kit (Genepharma, Shanghai, China) using RNU6B as an endogenous control. The data were processed using a 2^-ΔΔCT^ method.

### Luciferase reporter assay

The fragment of HRAS 3′-UTR or LINC00623 was amplified by PCR and cloned downstream of the Renilla gene in the psiCHECK2 vector (Promega, Madison, WI, USA), and the constructs were named wt-HRAS 3′-UTR or wt-LINC00623. To generate the HRAS 3′-UTR or LINC00623 mutant reporter, the miRNA binding site of the HRAS 3′-UTR or LINC00623 was mutated to remove the complementarity to miR-101, and the constructs were named mut-HRAS 3′-UTR or mut-LINC00623. HEK293 cells (ATCC, USA) were seeded into a 24-well plate. After culturing overnight, HEK293 cells were cotransfected with the indicated vectors and miR-101 mimics or miR-101 inhibitor. Luciferase assays were performed 48 h after transfection using the Dual Luciferase Reporter Assay System (Promega). Renilla luciferase activity was normalized to firefly luciferase activity for each transfected well.

### RNA immunoprecipitation (RIP)

RNA immunoprecipitation was performed using a Magna RIP RNA-Binding Protein Immunoprecipitation Kit (17-700, Millipore) with AGO2 antibody according to the manufacturer’s instructions. RNA for *in vitro* experiments was transcribed using a T7 High YieldRNA Synthesis Kit (E2040S, NEB) according to the manufacturer’s instructions. IgG, LINC00623 and miR-101 levels in the immunoprecipitates were measured by qRT-PCR.

### Statistical analysis

Data are processed using SPSS17.0 statistical software and presented as the mean ± S.D. of results from at least three independent experiments. A Student *t* test was used for statistical comparison between means where applicable. Differences among more than two groups in the above assays were estimated using one-way ANOVA. *P* < 0.05 was considered as statistically significant.

## Supplementary Material

Supplementary Figures

Supplementary Tables
