# Supplementary material for: LINC00623/miR-101/HRAS axis modulates IL-1β-mediated ECM degradation, apoptosis and senescence of osteoarthritis chondrocytes
Source: Aging (Albany NY). 2020 Feb 12;12(4):3218–37. doi: 10.18632/aging.102801 (PMC7066905; doi:10.18632/aging.102801)
Supplement: Supplementary Figures [file aging-12-102801-s002..pdf]

SUPPLEMENTARY FIGURES

Supplementary Figures

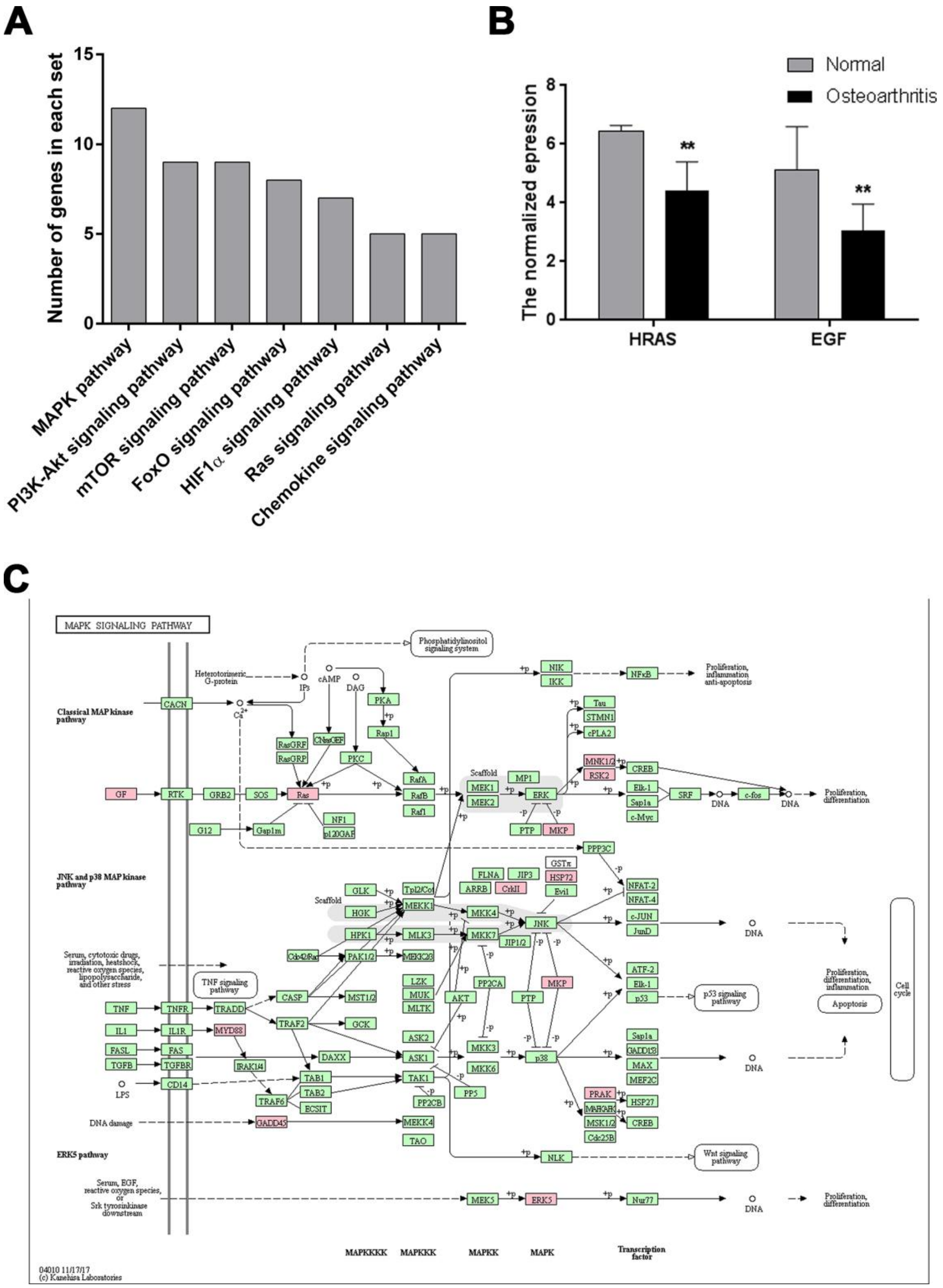

Supplementary Figure 1. The suppression of MAPK pathway in the osteoarthritis cartilage tissue. (**A**) Total of 566 significant down-regulated genes in osteoarthritis tissues were annotated and enriched in KEGG (Kyoto Encyclopedia of Genes and Genomes) pathway and the major altered cellular signaling pathways including MAPK, PI3K/AKT and mTOR pathway were shown. The number of genes in each set was also shown. (**B**) The relative expression of representative down-regulated MAPK pathway genes including HRAS(RAS) (Harvey rat sarcoma viral oncogene homolog) and EGF (Epidermal growth factor) were shown. (**C**) The altered crucial genes in MAPK the pathway (hsa04010).

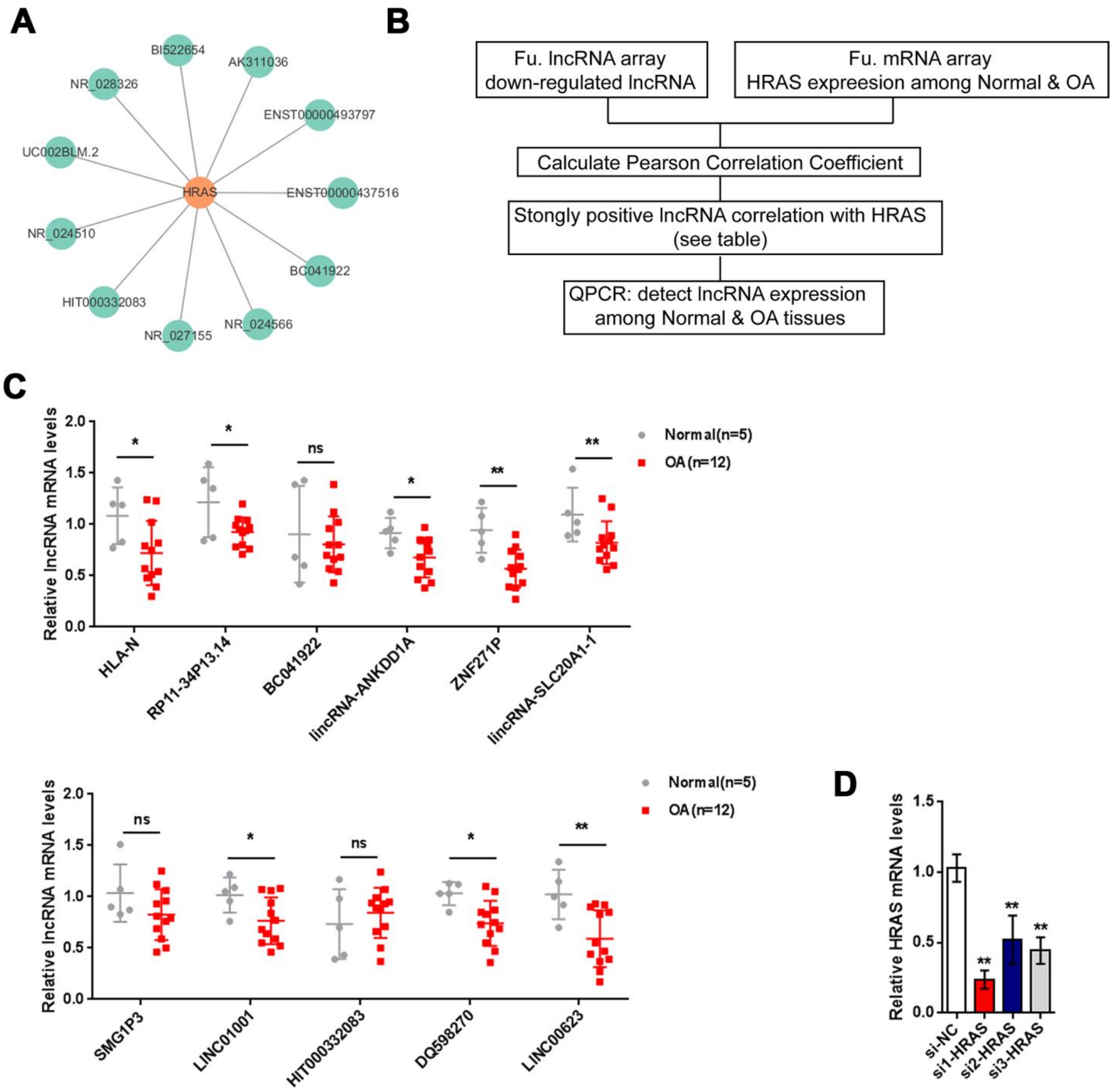

Supplementary Figure 2. Screening of lncRNAs related to HRAS. (**A**) a total of 11 lncRNAs were strongly positively correlated with HRAS, and LINC00623 was the most relevant one. (**B**) A schematic diagram showing the screening of lncRNAs correlated to HRAS. (**C**) A small sample size verification was performed to detect the expression of these lncRNAs in normal and OA tissues. (**D**) HRAS knockdown in OA chondrocytes was conducted by the transfection of si1-HRAS, si2-HRAS, or si3-HRAS; the transfection efficiency was verified by real-time PCR. **P*<0.05, ***P*<0.01.
