# Supplementary material for: LINC00623/miR-101/HRAS axis modulates IL-1β-mediated ECM degradation, apoptosis and senescence of osteoarthritis chondrocytes
Source: Aging (Albany NY). 2020 Feb 12;12(4):3218–37. doi: 10.18632/aging.102801 (PMC7066905; doi:10.18632/aging.102801)
Supplement: Supplementary Tables [file aging-12-102801-s001..pdf]

SUPPLEMENTARY TABLES

Supplementary Table 1. Enrichment analysis of KEGG pathway.

Category	Pathway name	Count	Proportion%	Enrichment P-Value
KEGG_PATHWAY	MAPK pathway	12	3.8	2.50E-04
KEGG_PATHWAY	PI3K-Akt signaling pathway	9	2.9	5.60E-04
KEGG_PATHWAY	mTOR signaling pathway	9	1.9	1.30E-03
KEGG_PATHWAY	FoxO signaling pathway	8	2.4	2.00E-03
KEGG_PATHWAY	HIF-1 signaling pathway	7	1.2	2.90E-02
KEGG_PATHWAY	Ras signaling pathway	5	1	3.20E-02
KEGG_PATHWAY	Chemokine signaling pathway	7	1.7	3.30E-02

Supplementary Table 2. LncRNAs correlated to HRAS.

LncRNA	Ensemble	Correlation efficient
ENST00000437516	HLA-N	0.901205548
ENST00000493797	RP11-34P13.14	0.905231127
BC041922		0.90593243
AK311036	lincRNA-ANKDD1A	0.910861733
NR_024566	ZNF271P	0.921351673
BI522654	lincRNA-SLC20A1-1	0.922856812
NR_027155	LOC100271836(SMG1P3)	0.927245685
NR_028326	LOC100133161(LINC01001)	0.93014676
HIT000332083		0.938368285
uc002blm.2	DQ598270	0.943346258
NR_024510	LOC728855(LINC00623)	0.953859386
